# Genomic Heterogeneity and Structural Variation in Soybean Near Isogenic Lines

**DOI:** 10.3389/fpls.2013.00104

**Published:** 2013-04-24

**Authors:** Adrian O. Stec, Pudota B. Bhaskar, Yung-Tsi Bolon, Rebecca Nolan, Randy C. Shoemaker, Carroll P. Vance, Robert M. Stupar

**Affiliations:** ^1^Department of Agronomy and Plant Genetics, University of MinnesotaSaint Paul, MN, USA; ^2^Department of Agronomy, Iowa State UniversityAmes, IA, USA; ^3^Corn Insects and Crop Genetics Research Unit, Agricultural Research Service, United States Department of AgricultureAmes, IA, USA; ^4^Plant Research Unit, Agricultural Research Service, United States Department of AgricultureSaint Paul, MN, USA

**Keywords:** soybean, NIL, CGH, iron, heterogeneity

## Abstract

Near isogenic lines (NILs) are a critical genetic resource for the soybean research community. The ability to identify and characterize the genes driving the phenotypic differences between NILs is limited by the degree to which differential genetic introgressions can be resolved. Furthermore, the genetic heterogeneity extant among NIL sub-lines is an unaddressed research topic that might have implications for how genomic and phenotypic data from NILs are utilized. In this study, a recently developed high-resolution comparative genomic hybridization (CGH) platform was used to investigate the structure and diversity of genetic introgressions in two classical soybean NIL populations, respectively varying in protein content and iron deficiency chlorosis (IDC) susceptibility. There were three objectives: assess the capacity for CGH to resolve genomic introgressions, identify introgressions that are heterogeneous among NIL sub-lines, and associate heterogeneous introgressions with susceptibility to IDC. Using the CGH approach, introgression boundaries were refined and previously unknown introgressions were revealed. Furthermore, heterogeneous introgressions were identified within seven sub-lines of the IDC NIL “IsoClark.” This included three distinct introgression haplotypes linked to the major iron susceptible locus on chromosome 03. A phenotypic assessment of the seven sub-lines did not reveal any differences in IDC susceptibility, indicating that the genetic heterogeneity among the lines does not have a significant impact on the primary NIL phenotype.

## Introduction

The soybean (*Glycine max*) research community has developed valuable new molecular and genomic resources in recent years. Foremost among these was the public release of the genome sequence, assembled from the reference cultivar “Williams 82” (Schmutz et al., 2010). The genome sequence facilitated the development of additional genomics platforms, including new simple sequence repeat (SSR) panels, single nucleotide polymorphism (SNP) arrays, gene expression arrays, and resequencing-based genotyping methodologies (Hyten et al., 2010; Lam et al., 2010; Song et al., 2010; Varala et al., 2011; Le et al., 2012). While most of the developments have focused on high-throughput resolution of single nucleotide changes, a comparative genomic hybridization (CGH) microarray was also developed to detect larger structural genomic changes, such as deletions and duplications. This CGH platform has been utilized for detecting natural and induced genomic variants for a sub-set of interesting soybean accessions and mutant lines (Bolon et al., 2011; Haun et al., 2011; McHale et al., 2012).

Just as the genomic tools available to soybean researchers have been rapidly expanding in recent years, the expansion of soybean genetic resources has continued, but at a more modest rate. As such, the newer genomic tools have been used to address long-standing questions that are relevant to the community (i.e., enabling researchers to reassess old questions in new ways). One of the great long-standing genetic resources available to the soybean community is a large collection of near isogenic lines (NILs), developed decades ago, that exhibit variation for an extensive suite of traits (Bernard, 1975; Bernard et al., 1991).

Previous work on the physical mapping of genetic introgressions in soybean NILs found that expression arrays, SNP panels, and resequencing approaches were complementary in resolving the introgression boundaries (Severin et al., 2010). More specifically, Severin et al. (2010) identified seven introgression differences between cultivar “Clark” and its NIL “IsoClark.” “IsoClark” was developed by backcrossing susceptibility to iron deficiency chlorosis (IDC) traits from the donor line “T203” into the “Clark” genetic background. In this study, we were interested in adding another layer to this analysis, by exploring the genomic structural variants that are introgressed in soybean NIL stocks. Previous work in maize used CGH data on a panel of NILs to discover and validate quantitative trait loci (QTL) for plant height variation (Eichten et al., 2011), thereby demonstrating that structural variants can be used as high-resolution genetic markers for introgression mapping. Our first goal in this study was to use CGH polymorphisms as genetic markers to better resolve soybean NIL introgression boundaries, in both the “Clark” – “IsoClark” IDC NIL pair and a distinct seed protein NIL pair. Furthermore, we sought to identify genetic heterogeneity among “IsoClark” sub-lines and explore the possible relationship between sub-line variation and IDC susceptibility.

## Results

### High-resolution mapping of genomic introgressions in seed protein near isogenic lines

The HiPro and LoPro NILs were derived by introgressing a portion of the *Glycine soja* genome into a soybean background. As their names suggest, the HiPro line exhibits a higher seed protein content than the LoPro line (Nichols et al., 2006; Bolon et al., 2010). These lines have been previously genotyped using SNP and resequencing approaches, identifying large differential introgressions on chromosomes 18 and 20, and a small differential introgression on chromosome 16 (Severin et al., 2010). Since the introgressions are donated from *G. soja*, we reasoned that they would exhibit profound structural variation compared to *Glycine max* at these loci, and may reveal introgressions that have not been identified to date.

**Figure 1:**
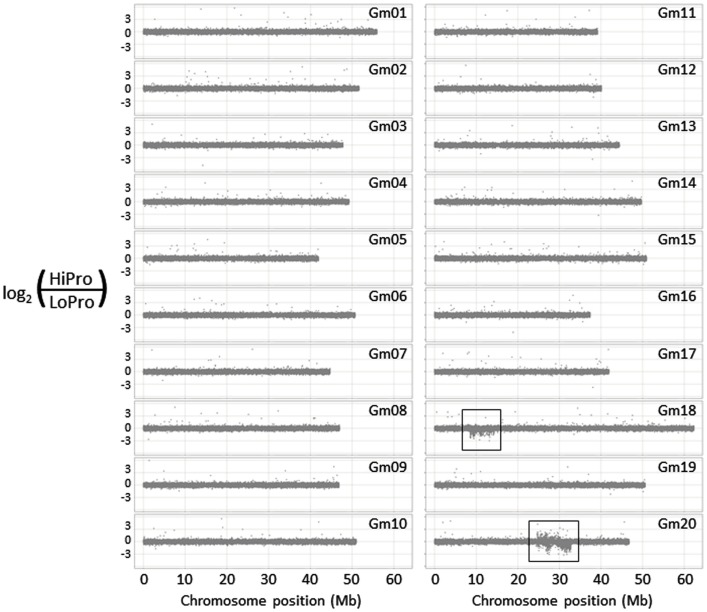
**Structural genomic analysis of the HiPro versus LoPro near isogenic lines**. Two conspicuous polymorphic regions are visible on chromosomes 18 and 20 (boxed regions), corresponding to previously identified loci.

We used the 700-k CGH microarray (Haun et al., 2011) to profile the structural difference between HiPro and LoPro (Figure 1). While the introgression on chromosome 16 was not detected using this approach (it is estimated to be only ∼10 kb), the differential introgressions on chromosomes 18 and 20 were clearly visible. Furthermore, the density of the polymorphic features in these regions allowed for a higher resolution estimate of the introgression positions than have been previously obtained. In effect, the CGH data expanded the estimated introgression regions by 1.20 and 1.74 Mb, respectively (Table 1). These findings indicated that CGH analyses can be used to further resolve the genomic positioning and structure of donor introgressions.

**Table 1:** **Comparison of differential *G. soja* introgression sites among the HiPro and LoPro NILs**.

Chromosome no.	Positions of previously identified introgressions*	Introgressions identified with CGH	Comments
Gm16	35581397–35591171	None	Estimated introgression: 0.009 Mb; not found with CGH
Gm18	8828934–13426557	8541756–14338302	Estimated introgression: 5.80 Mb; CGH extended boundaries
Gm20	26485526–32766318	24722474–32747999	Estimated introgression: 8.03 Mb; CGH extended boundaries

*Introgressions identified with RNA-Seq data (Severin et al., 2010). Nucleotide positions are based on the soybean reference genome sequence (assembly version 1.01) (Schmutz et al., 2010).

### Diversity of genomic introgressions for sub-lines of iron deficiency chlorosis NILs

The iron susceptible genotype “T203” was previously backcrossed into the iron-tolerant “Clark” genetic background to form a NIL, called “IsoClark,” with enhanced iron susceptibility. The “Clark” – “IsoClark” NIL pair has been extensively characterized at the genomic, transcriptomic, and phenotypic levels to elucidate the genetic mechanisms that underlie IDC. However, all studies to date have presented the analysis of a single “IsoClark” line, without considering the possibility that genetically distinct sub-lines may exist within the “IsoClark” population.

We used CGH with an updated 1.3-million feature long-oligo microarray to scan the genetic differences that may be present among seven “IsoClark” individuals (the seven sub-lines were renamed IsoClark 1, IsoClark 2, and so on). “Clark” was labeled as the common Cy5 reference in each experiment, with each “IsoClark” individual labeled with Cy3. The “T203” parental line was also hybridized with the “Clark” reference to determine the structural genomic differences between the parental lines. “T203” introgressions in “IsoClark” individuals were identified on chromosomes 03, 04, 05, 08, 09, 13, 14, and 16 (Table 2). While most of these introgressions were previously found using other methods, this was the first identification of the chromosome 09 and 14 introgressions. Furthermore, the mapped regions for several of the introgression boundaries were better resolved using the CGH method (Table 2).

**Table 2:** **Comparison of introgression sites and CNV boundaries among IsoClark/Clark lines using CGH and other approaches**.

Chromosome no.	Positions of previously identified introgressions*	Introgressions identified with CGH	Comments
Gm01	None	None	
Gm02	None	None	
Gm03	36398914–45743871	36382323–45532330**	Estimated introgression: 9.36 Mb
Gm04	44751336–45597626	44736519–45611892	Estimated introgression: 0.88 Mb
Gm05	38251772–39085416	38240630–38973056	Estimated introgression: 0.84 Mb
Gm06	None	None	
Gm07	None	None	
Gm08 (top)	2040000–3060000	None	Estimated introgression: 1.02 Mb; not found with CGH
Gm08 (bottom)	43883437–46941690	43779981–46965555	Estimated introgression: 3.19 Mb
Gm09	None	12714220–30892739***	Estimated introgression: 18.2 Mb; found only by CGH
Gm10	None	None	
Gm11	None	None	
Gm12	None	None	
Gm13	35524268–35862205	35521785–35876466	Estimated introgression: 0.35 Mb
Gm14	None	17302467–17306627***	Estimated introgression: 0.004 Mb; found only by CGH
Gm15	None	None	
Gm16	30464934–31885123	30469633–31905827	Estimated introgression: 1.44 Mb
Gm17	None	None	
Gm18	None	None	
Gm19	None	None	
Gm20	None	None	

*Introgressions identified with RNA-Seq/SFP/GoldenGate on a single sub-line of IsoClark (Severin et al., 2010).

**Figure 2:**
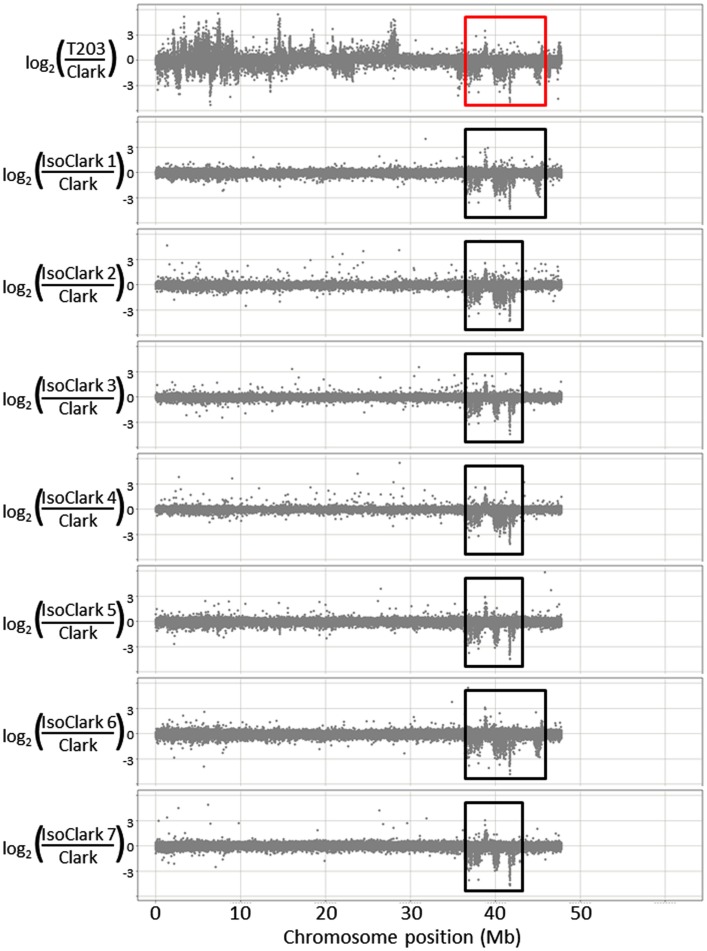
**Genomic structural variation on chromosome 03 for seven different “IsoClark” NILs**.

**Positions of the chromosome 3 “Type 1” introgression (see Figure 2).

***Newly identified introgression in this study.

The seven different “IsoClark” sub-lines exhibited some chromosomes with uniformity among the individuals, and some chromosomes with differential introgressions among the individuals (Table 3). All seven “IsoClark” sub-lines exhibited introgressions on chromosomes 04, 13, 14, and 16, while only a sub-set exhibited introgressions on chromosomes 05, 08, and 09 (Figure S1 in Supplementary Material). Only two “IsoClark” sub-lines (#1 and #6) exhibited identical introgression profiles, indicating that there are at least six genetically distinct “IsoClark” sub-lines in the population.

**Table 3:** **Frequency and presence of introgressed regions within seven sub-lines of IsoClark**.

Chromosome no.	IsoClark #1	IsoClark #2	IsoClark #3	IsoClark #4	IsoClark #5	IsoClark #6	IsoClark #7
Gm03*	Yes (Type 1)	Yes (Type 2)	Yes (Type 3)	Yes (Type 2)	Yes (Type 3)	Yes (Type 1)	Yes (Type 3)
Gm04	Yes	Yes	Yes	Yes	Yes	Yes	Yes
Gm05	Yes	Yes	No	Yes	No	Yes	No
Gm08 (Bottom)	Yes	No	Yes	No	Yes	Yes	Yes
Gm09	No	Yes	Yes	No	Yes	No	Yes
Gm13	Yes	Yes	Yes	Yes	Yes	Yes	Yes
Gm14	Yes	Yes	Yes	Yes	Yes	Yes	Yes
Gm16	Yes	Yes	Yes	Yes	Yes	Yes	Yes

**Figure 3:**
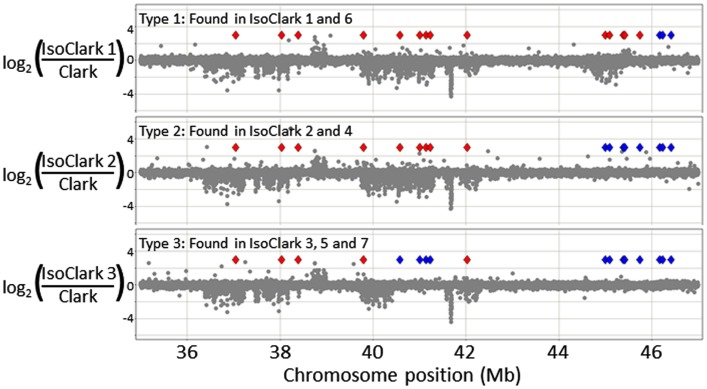
**A detailed view of thee different “IsoClark” introgressions on chromosome 03**. The CGH data is shown as gray spots. Diamonds represent the positions of SNP markers in this region: red diamonds indicate SNPs matching the “T203” haplotype, while blue diamonds indicate SNPs matching the “Clark” haplotype. As expected, the introgressed “T203” regions exhibit structural variation (UpCNV and DownCNV) relative to the “Clark” control. Haplotype 1 (top) appears to be a continuous ∼10 Mb introgression of “T203,” haplotype 2 appears to be a smaller (∼6–8 Mb) introgression, and haplotype three appears to be a quadruple recombinant where part of the introgression is interrupted by the “Clark” haplotype (at position ∼41 Mb).

*The chromosome 03 introgression has three different types (see Figure 3).

Particularly interesting introgression patterns were observed on chromosome 03. While all seven “IsoClark” sub-lines exhibited introgressions on chromosome 03, three different forms were observed (Figure 2). Two sub-lines (#1 and #6) exhibited a substantially larger introgression that the others. Furthermore, the smaller introgression was found to be either continuous (#2 and #4) or interrupted (#3, #5, and #7) by the “Clark” haplotype around position 41 Mb. The interruption of the “Clark” DNA, presumably caused by additional recombination events within this haplotype, was further confirmed by SNP genotyping calls on these sub-lines (Figure 3).

### Phenotypic assessment of iron deficiency traits in the different “IsoClark” sub-lines

**Figure 4:**
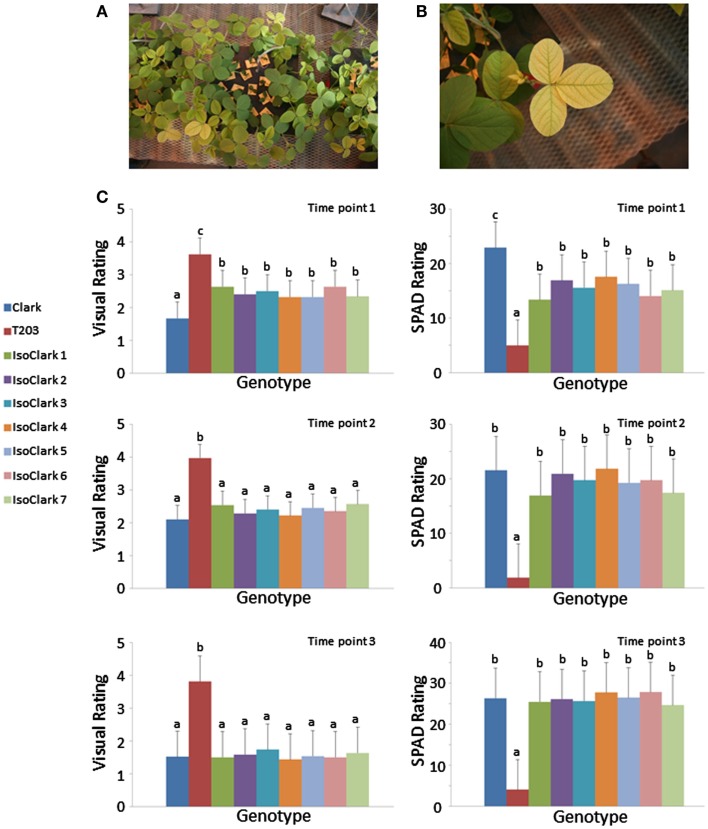
**Yellowing responses of the “IsoClark” sub-lines to hydroponic treatments of limited iron**. **(A)** All nine genotypes (including “Clark” and “T203” control) were grown together within each treatment unit. **(B)** Conspicuous yellowing of a “T203” trifoliate. **(C)** Mean separation (LSD) for phenotypes as scored by visual ratings and SPAD measurements over three time points. Letters a, b, c, indicate when treatment means are in significantly different groups.

The “IsoClark” sub-lines displayed genetic heterogeneity on some chromosomes, posing the question of whether the different “T203” introgressions result in differences in IDC susceptibility. We grew the seven “IsoClark” sub-lines, along with the “Clark” and “T203” controls, in the greenhouse under hydroponic conditions to test for IDC responses under limited iron conditions as previously described (O’Rourke et al., 2007). Differential yellowing in the foliage was clearly observed among the plants (Figures 4A,B). Yellowing was scored both visually and with a SPAD meter at three different time points early in development (Figure 4C). As expected, the “IsoClark” sub-lines exhibited more yellowing than “Clark,” but less yellowing than “T203,” during the earliest developmental stage. In the subsequent two time points, the “IsoClark” sub-lines exhibited a phenotype similar to “Clark,” while “T203” continued to show the greatest degree of yellowing. Importantly, our data indicate that the seven “IsoClark” sub-lines were similar across all time points using both the visual and SPAD measurements. The different genetic introgressions among the seven sub-lines apparently did not have a major influence on the IDC phenotype, suggesting that the introgressed susceptibility loci are present in all seven “IsoClark” individuals.

## Discussion

### Technical considerations: Resolution and newly discovered introgressions in soybean NILs

The best previous assessments of genetic introgression in the soybean “Clark” – “IsoClark” isolines relied on SNP-based platforms, ranging from a pre-ascertained set of 1,536 markers to an RNA-Seq approach which identified hundreds of markers specifically within the introgressed regions (Severin et al., 2010). It has been shown that a combination of the different marker systems complemented one another, resulting in increased resolution of introgression events and boundaries. In this study, we expand upon this finding, as the CGH microarray platform identified introgressions that had previously not been detected in “IsoClark,” and further resolved the introgression boundaries of the known introgressions in “IsoClark” and the HiPro – LoPro NIL pair.

Most strikingly, the CGH analysis revealed a putative ∼18.2 Mb introgression on “IsoClark” chromosome 09 that had been previously undetected. At first glance, it may seem improbable that such a large introgression would have previously escaped detection. However, the chromosome 09 introgression is heterogeneous among “IsoClark” sub-lines. Furthermore, the previous study that most aggressively genotyped “IsoClark” (Severin et al., 2010) utilized a sub-line (renamed here as IsoClark 1) that lacked this particular introgression. However, assaying seven sub-lines with a high density 1.3-million feature CGH platform revealed that this introgression is found in a sub-set of the “IsoClark” individuals.

One other previously undetected “IsoClark” introgression event, on chromosome 14, was detected in this study. The situation at this locus is very different than the chromosome 09 locus. The chromosome 09 introgression is large and heterogeneous among sub-lines, while the chromosome 14 introgression appears to be small and homogenous (always present) among the sub-lines, exhibiting a prominent single CGH peak. It is not surprising that this event has been previously undetected, due to the small size of the introgression. However, as stated above, the high density of the CGH microarray enabled this discovery. These data further confirm the value of using high density marker platforms to detect genetic polymorphisms that are generally recalcitrant to identification.

### Genetic considerations: Implications for gene discovery in the IDC NILs

It has long been known from mapping studies using multiple populations that a major QTL for IDC maps to soybean chromosome 03 (Lin et al., 1997, 2000). The “Clark” – “IsoClark” NIL pair has been a model for dissecting the genetic and molecular variation that is associated with this chromosome 03 QTL. While the physical mapping of the “T203” genetic introgression has provided a roadmap for this locus (Severin et al., 2010), transcriptome-based studies have provided further insights into the gene expression reprogramming that accompanies the IDC differences in the NIL pair (O’Rourke et al., 2007, 2009). The introgression and transcription data suggest that the IDC difference may be conferred by a transcription factor within the chromosome 03 introgression. Furthermore, recent genetic fine-mapping of the “Clark” – “IsoClark” NIL pair indicates that the major QTL conferring the differential IDC responses in this material resides within a specific region of the chromosome 03 introgression (Peiffer et al., 2012). Peiffer et al. (2012) recently proposed that the IDC intolerance of certain soybean lines (including “T203” and “IsoClark”) may be caused by a 12-bp deletion in the second exon of a Fe-DEFICIENCY-INDUCED TRANSCRIPTION FACTOR (Glyma03g28610) that affects the induction of iron acquisition genes.

While the seven “IsoClark” sub-lines used in this study revealed three different chromosome 03 haplotypes, all forms included the mapped IDC locus and the accompanying candidate gene. Furthermore, while it was tempting to hypothesize that the genetic background differences between the seven sub-lines may influence the IDC trait, the phenotyping analyses performed in this study did not identify any statistical difference between the seven individuals. Therefore, our data offer further evidence that the major “IsoClark” IDC QTL may be conferred by this single chromosome 03 locus, and perhaps by a single gene.

## Materials and Methods

### Plant materials

Two sets of soybean (*Glycine max*) NILs were obtained for comparative analyses. The HiPro – LoPro NIL pair was derived from introgressing *G. soja* (PI468916) DNA into soybean (A81-356022) and has been previously described (Nichols et al., 2006; Bolon et al., 2010; Severin et al., 2010). The IDC “IsoClark” NIL (PI 547430) was derived from backcrossing an iron susceptible locus from soybean line “T203” (PI 54619) into the “Clark” (PI 548533) genetic background (Bernard et al., 1991). Four of the seven “IsoClark” sub-lines (#1, #2, #3, and #6) used for this study were derived from a single packet of seed obtained from Iowa State University, which had been previously harvested from a bulked seed increase. The remaining three “IsoClark” sub-lines were derived from seeds harvested from a bulked seed increase at the University of Minnesota in 2009 and 2010; however, the original source of these sub-lines is undocumented. The “Clark,” “T203” genotypes, and the IsoClark 1 sub-line were used for genotyping in a previous study (Severin et al., 2010).

To prepare the samples for CGH analysis, seeds for the above described lines were planted in individual 4′′ pots containing a 50:50 mix of sterilized soil and Metro Mix and grown under standard greenhouse conditions. Young trifoliate leaves from 3-week-old plants were harvested and immediately frozen in liquid nitrogen. Frozen leaf tissue was ground with a mortar and pestle with liquid nitrogen. DNA was extracted from ∼100 mg of ground tissue using the Qiagen Plant DNeasy Mini Kit according to the manufacturer’s protocol (including an RNA degradation step). DNA was quantified on a NanoDrop spectrophotometer.

### Comparative genomic hybridization and SNP data analysis

Comparative genomic hybridization for the HiPro – LoPro comparison was performed as described (Haun et al., 2011) on the NimbleGen soybean CGH 700 k microarray, which consists of 696,139 unique oligonucleotide probes (50–75 m) designed from the reference “Williams 82” sequence (assembly version 1.01) (Schmutz et al., 2010) and placed at ∼1.1 kb intervals. The HiPro individual was labeled with Cy3 and the LoPro individual was labeled with Cy5. The segMNT algorithm in the NimbleScan software (version 2.5) was used to extract the raw data and make segmentation calls. The parameters of the algorithm were as follows: minimum segment difference = 0.1, minimum segment length (number of probes) = 2, acceptance percentile = 0.99, number of permutations = 10, non-unique probes were included, and spatial correction and qspline normalization were applied. The log_2_ ratio between the HiPro and LoPro signal for each probe were computed and visual displays of the CGH data were generated using Spotfire DecisionSite software. An approximation of the introgression boundaries were inferred from visual examination of the log_2_ plots.

The “Clark” – “IsoClark” CGH experiments were conducted on an updated 1.3-million feature NimbleGen soybean CGH microarray. This microarray consists of 1,344,283 unique oligonucleotide probes (50–75 nucleotides) also designed from the reference soybean genome sequence (assembly version 1.01) (Schmutz et al., 2010). The probes are placed at a median interval of ∼500 bp between probes. The “T203” and “IsoClark” individuals were labeled with Cy3 and the “Clark” individual was labeled with Cy5 (the Cy5 “Clark” samples served as the common reference for all hybridizations). The segMNT algorithm in the NimbleScan software (version 2.5) was used to extract the raw data and make segmentation calls, with the same parameters as described above for the HiPro – LoPro comparison. The log_2_ ratio between the Cy3 genotype and “Clark” were computed and visual displays of the CGH data were generated using Spotfire DecisionSite software. An approximation of the introgression boundaries were inferred from visual examination of the log_2_ plots.

Genotyping of “Clark,” “T203,” and the seven “IsoClark” sub-lines was performed using the Illumina 1,536 SNP platform for soybean (Hyten et al., 2010). The DNA samples were processed at the University of Minnesota BioMedical Genomics Center.

### Phenotyping of IDC traits in hydroponic conditions

Iron deficiency chlorosis phenotyping in iron limited conditions essentially used the hydroponic methodology that has been previously described (O’Rourke et al., 2007; Peiffer et al., 2012). Briefly, the seeds were started on moist germination paper and transferred to hydroponic solutions after 6 days. The plants were grown in 10 L buckets under greenhouse conditions in an iron deficient hydroponic solution containing 50 μm Fe(NO_3_)_3_·9H_2_O (the full hydroponic formulation and detailed growth conditions are provided in previously published work (Peiffer et al., 2012)). The greenhouse photoperiod was 16 h of light and 8 h of dark. Each of six buckets contained 18 plants, with two technical replicates for each genotype (“Clark,” “T203,” and “IsoClark” sub-lines 1–7). Foliage color was scored visually and with a SPAD meter at three different time points. The visual scoring system was based on a previously defined 1–5 scale (Cianzio et al., 1979), and examples of the phenotypic distribution along this spectrum are shown in Figure S2 in Supplementary Material. For Time point 1, the first trifoliate of each plant was scored following 14 days in the hydroponic conditions. For time point 2, the second trifoliate was scored following 18 days in the hydroponic conditions. For Time point 3, the third trifoliate was scored following 22 days in the hydroponic conditions.

For statistical analysis, the two technical replicates per genotype per bucket were averaged to give a replicate value. Therefore, the six buckets were treated as biological replicates for each genotype. We did an Analysis of Variance (*Y*_i,j_ = μ + Genotype_i_ + ε) to detect significant genotype effects and Fisher’s Least Significant Difference analysis to identify significant differences between individual genotypes.

### Accession numbers

The CGH data from this study have been submitted to the National Center for Biotechnology Information Gene Expression Omnibus (http://www.ncbi.nlm.nih.gov/geo). The comparison involving HiPro and LoPro can be found as accession number GSE44725 and the comparisons involving “Clark,” “T203,” and the “IsoClark” sub-lines are under accession number GSE44789.

## Conflict of Interest Statement

The authors declare that the research was conducted in the absence of any commercial or financial relationships that could be construed as a potential conflict of interest.

## Supplementary Material

The Supplementary Material for this article can be found online at http://www.frontiersin.org/Plant_Genetics_and_Genomics/10.3389/fpls.2013.00104/abstract

Supplementary Figure S1 **“T203” introgressions in seven “IsoClark” sub-lines on chromosomes 04, 05, 08, 09, 13, 14, and 16**.

Supplementary Figure S2 **Examples of the 1–5 scale used for the visual scoring of IDC in the hydroponic treatments**.
