# Supplementary material for: Genomic Heterogeneity and Structural Variation in Soybean Near Isogenic Lines
Source: Front Plant Sci. 2013 Apr 24;4:104. doi: 10.3389/fpls.2013.00104 (PMC3633938; doi:10.3389/fpls.2013.00104)
Supplement: Supplementary Figure S1 — “T203” introgressions in seven “IsoClark” sub-lines on chromosomes 04, 05, 08, 09, 13, 14, and 16. [file Presentation_1.ppt]

## Slide 1
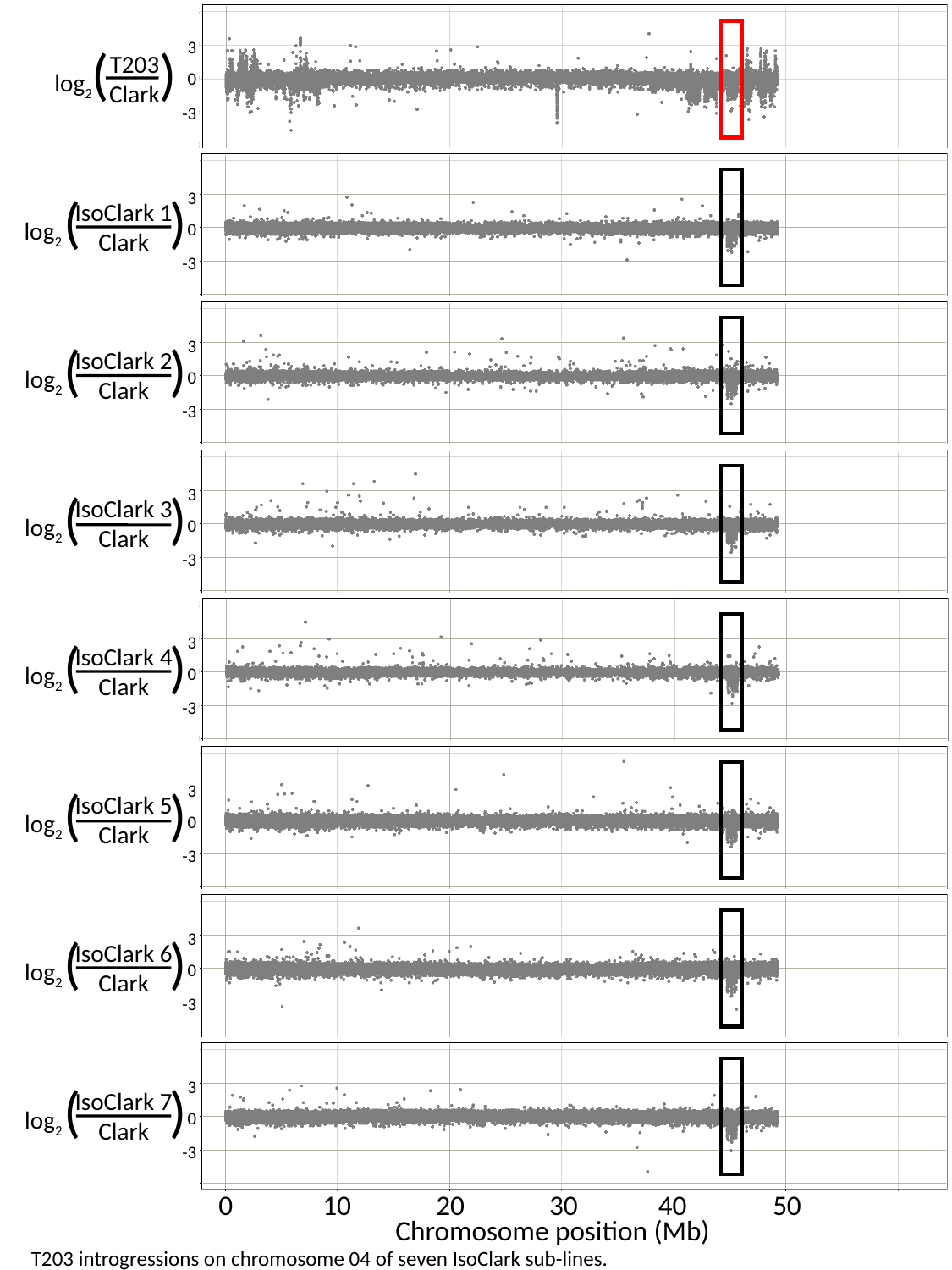

3
( )
T203
Clark
log2
0
-3
( )
3
IsoClark 1
Clark
log2
0
-3
( )
3
IsoClark 2
Clark
log2
0
-3
( )
3
IsoClark 3
Clark
log2
0
-3
( )
3
IsoClark 4
Clark
log2
0
-3
( )
3
IsoClark 5
Clark
log2
0
-3
( )
3
IsoClark 6
Clark
log2
0
-3
( )
3
IsoClark 7
Clark
log2
0
-3
0
10
20
30
40
50
Chromosome position (Mb)
T203 introgressions on chromosome 04 of seven IsoClark sub-lines.

## Slide 2
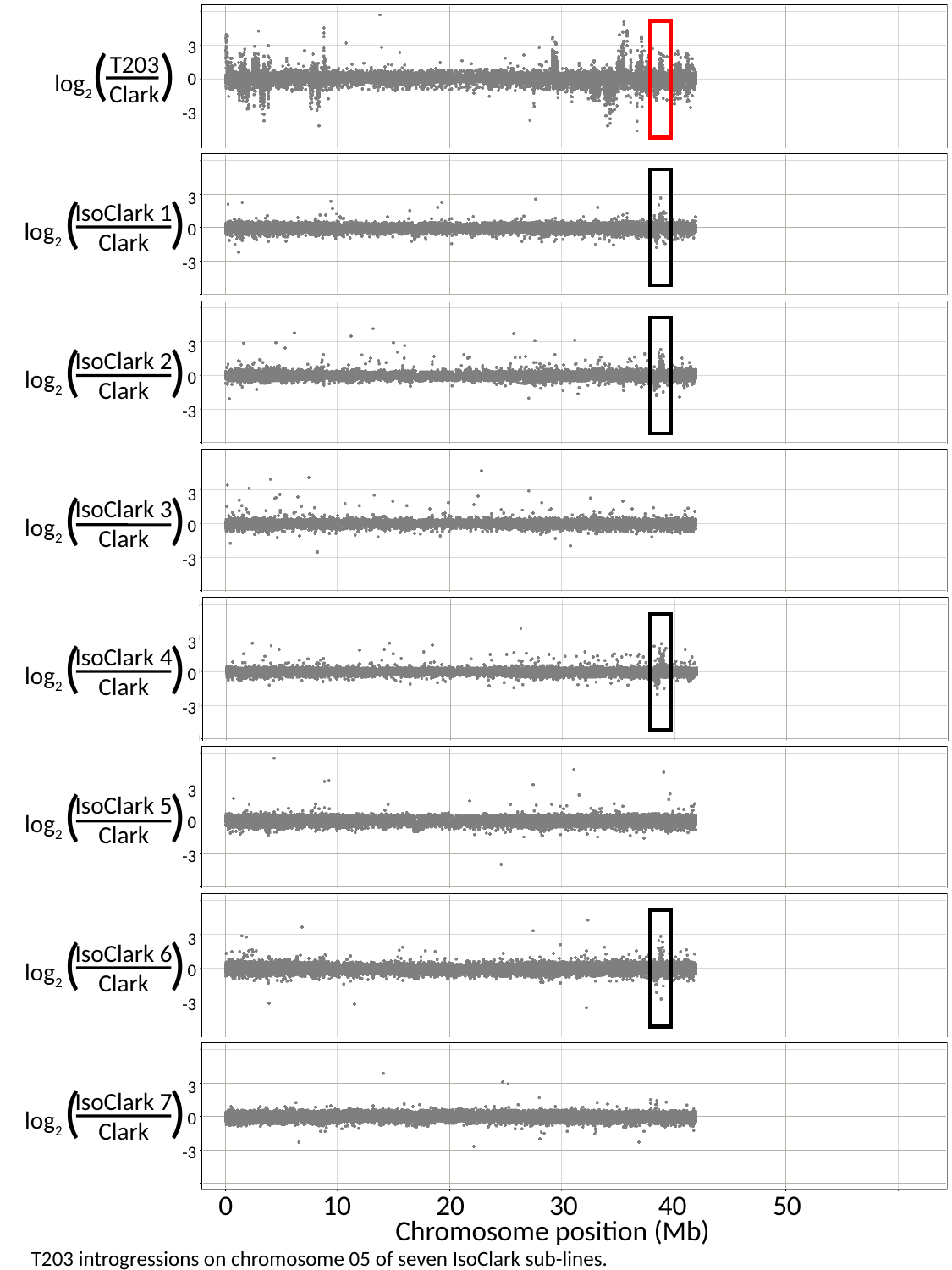

3
( )
T203
Clark
log2
0
-3
( )
3
IsoClark 1
Clark
log2
0
-3
( )
3
IsoClark 2
Clark
log2
0
-3
( )
3
IsoClark 3
Clark
log2
0
-3
( )
3
IsoClark 4
Clark
log2
0
-3
( )
3
IsoClark 5
Clark
log2
0
-3
( )
3
IsoClark 6
Clark
log2
0
-3
( )
3
IsoClark 7
Clark
log2
0
-3
0
10
20
30
40
50
Chromosome position (Mb)
T203 introgressions on chromosome 05 of seven IsoClark sub-lines.

## Slide 3
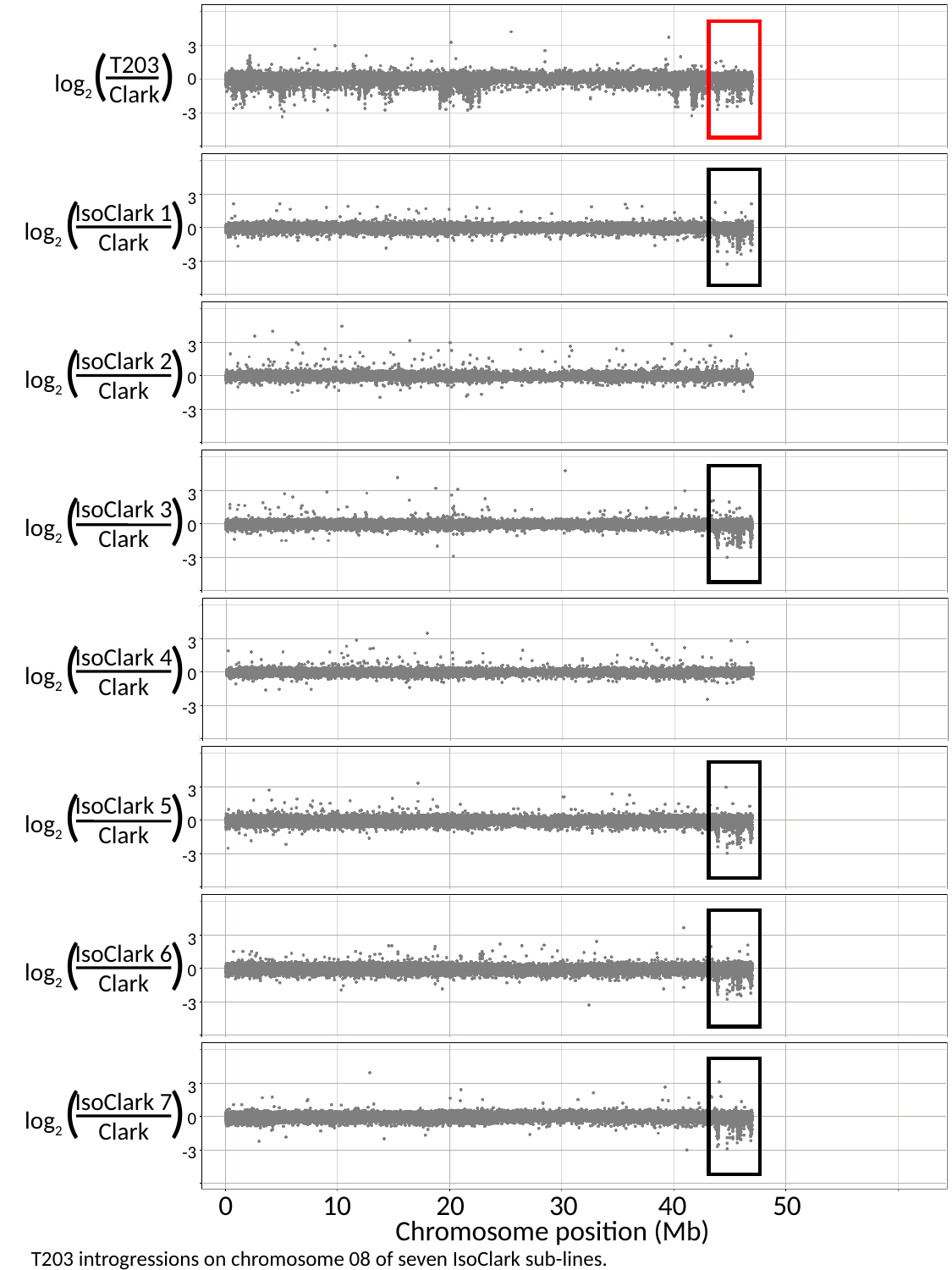

3
( )
T203
Clark
log2
0
-3
( )
3
IsoClark 1
Clark
log2
0
-3
( )
3
IsoClark 2
Clark
log2
0
-3
( )
3
IsoClark 3
Clark
log2
0
-3
( )
3
IsoClark 4
Clark
log2
0
-3
( )
3
IsoClark 5
Clark
log2
0
-3
( )
3
IsoClark 6
Clark
log2
0
-3
( )
3
IsoClark 7
Clark
log2
0
-3
0
10
20
30
40
50
Chromosome position (Mb)
T203 introgressions on chromosome 08 of seven IsoClark sub-lines.

## Slide 4
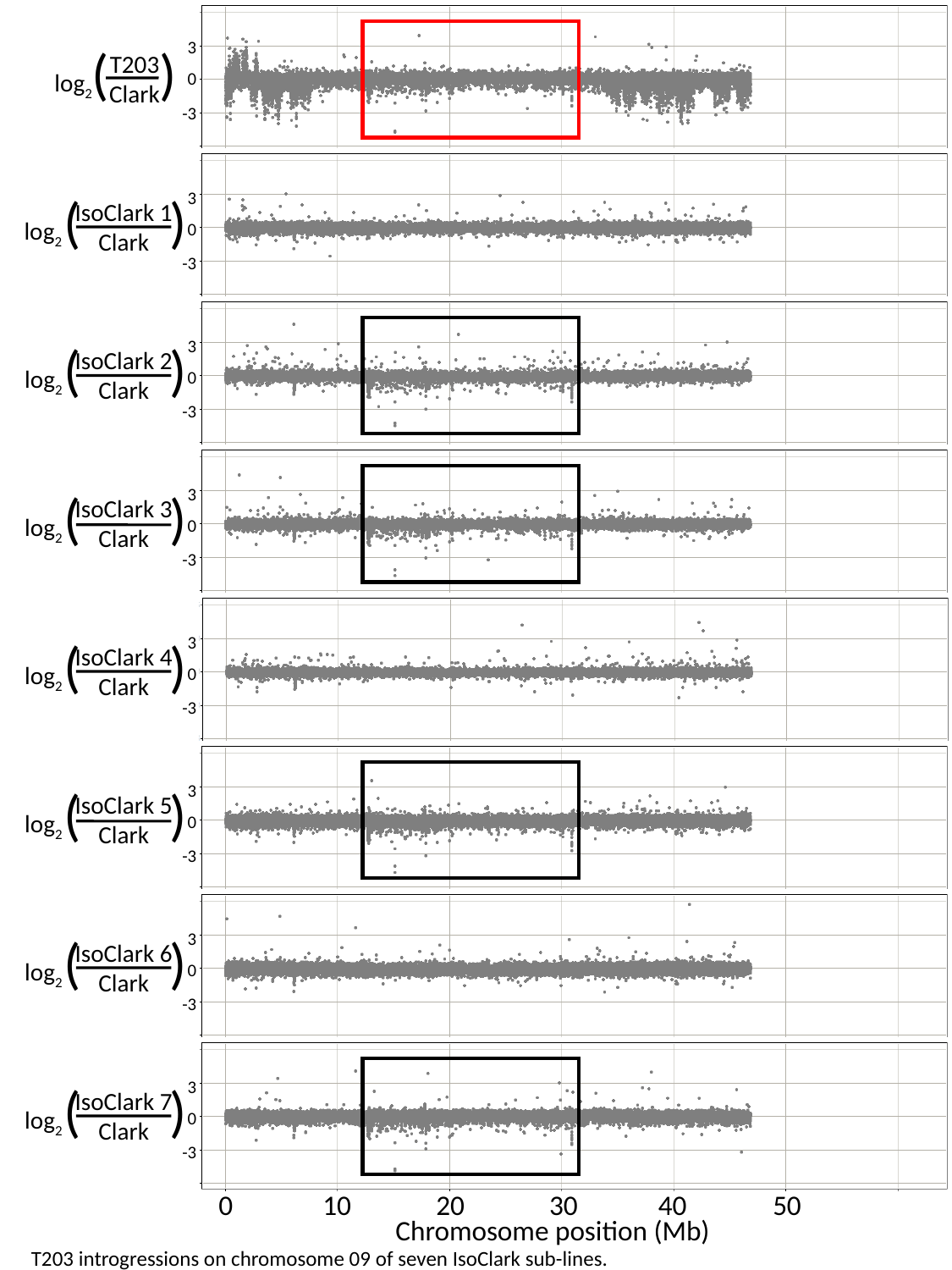

3
( )
T203
Clark
log2
0
-3
( )
3
IsoClark 1
Clark
log2
0
-3
( )
3
IsoClark 2
Clark
log2
0
-3
( )
3
IsoClark 3
Clark
log2
0
-3
( )
3
IsoClark 4
Clark
log2
0
-3
( )
3
IsoClark 5
Clark
log2
0
-3
( )
3
IsoClark 6
Clark
log2
0
-3
( )
3
IsoClark 7
Clark
log2
0
-3
0
10
20
30
40
50
Chromosome position (Mb)
T203 introgressions on chromosome 09 of seven IsoClark sub-lines.

## Slide 5
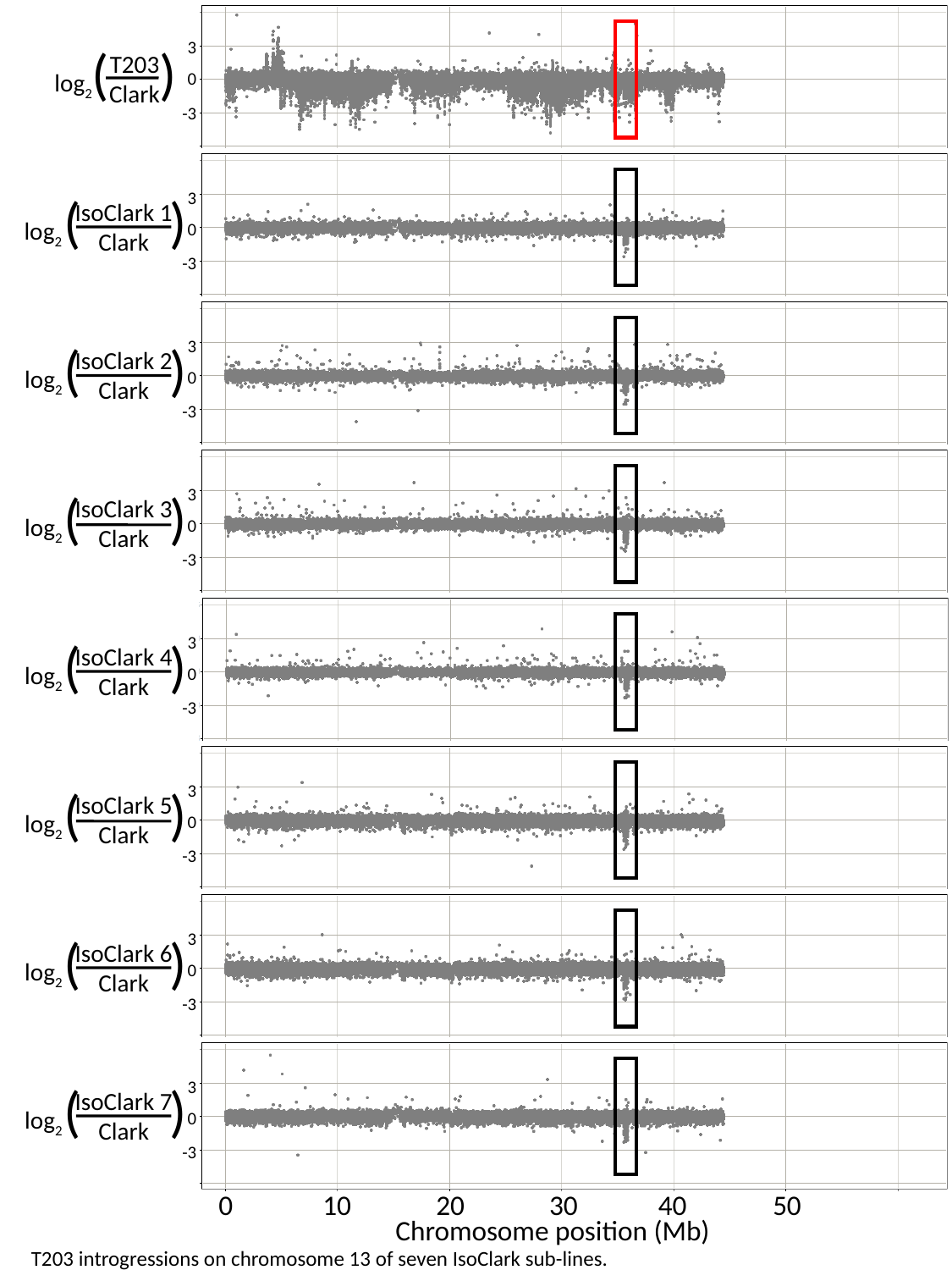

3
( )
T203
Clark
log2
0
-3
( )
3
IsoClark 1
Clark
log2
0
-3
( )
3
IsoClark 2
Clark
log2
0
-3
( )
3
IsoClark 3
Clark
log2
0
-3
( )
3
IsoClark 4
Clark
log2
0
-3
( )
3
IsoClark 5
Clark
log2
0
-3
( )
3
IsoClark 6
Clark
log2
0
-3
( )
3
IsoClark 7
Clark
log2
0
-3
0
10
20
30
40
50
Chromosome position (Mb)
T203 introgressions on chromosome 13 of seven IsoClark sub-lines.

## Slide 6
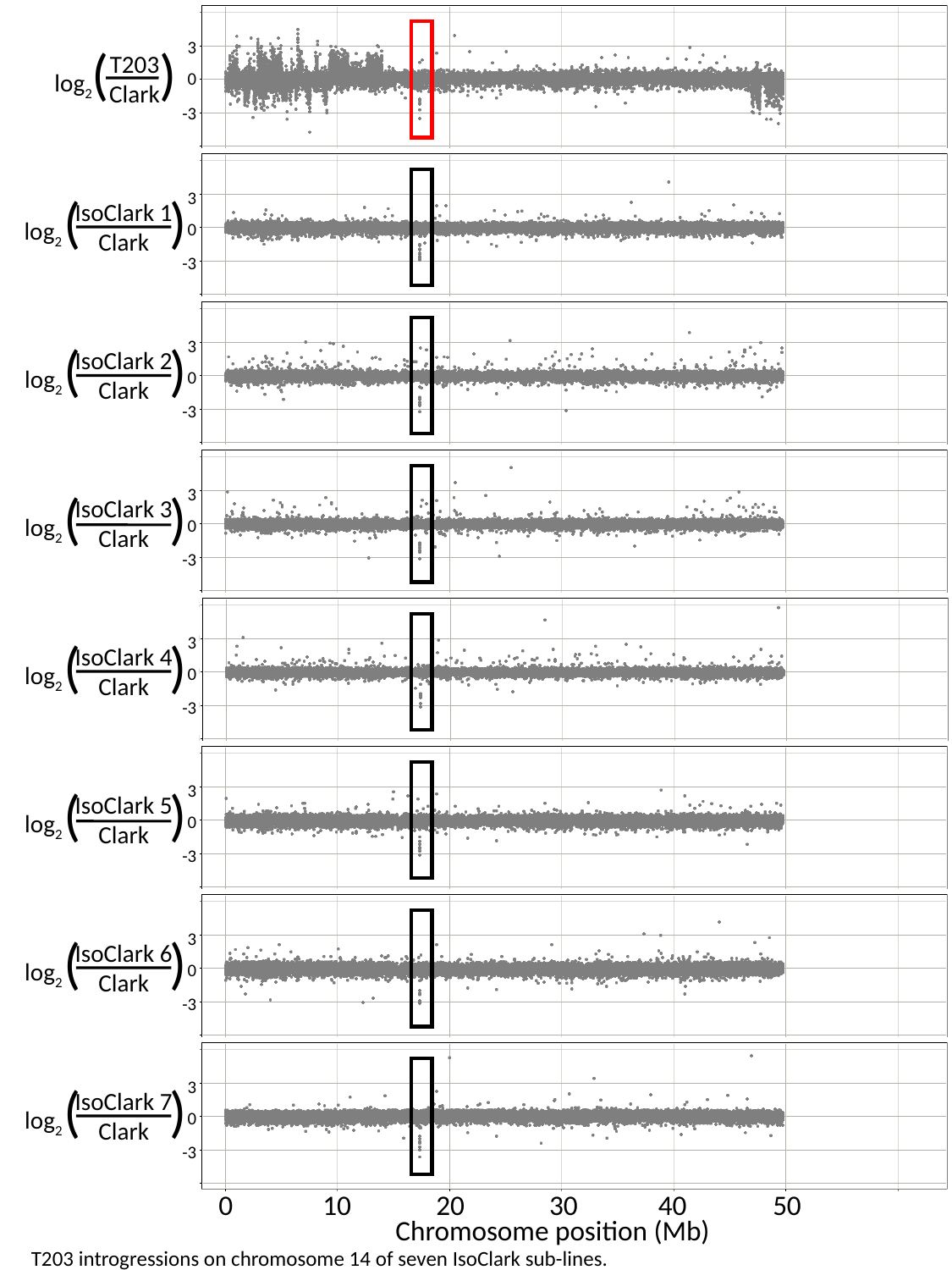

3
( )
T203
Clark
log2
0
-3
( )
3
IsoClark 1
Clark
log2
0
-3
( )
3
IsoClark 2
Clark
log2
0
-3
( )
3
IsoClark 3
Clark
log2
0
-3
( )
3
IsoClark 4
Clark
log2
0
-3
( )
3
IsoClark 5
Clark
log2
0
-3
( )
3
IsoClark 6
Clark
log2
0
-3
( )
3
IsoClark 7
Clark
log2
0
-3
0
10
20
30
40
50
Chromosome position (Mb)
T203 introgressions on chromosome 14 of seven IsoClark sub-lines.

## Slide 7
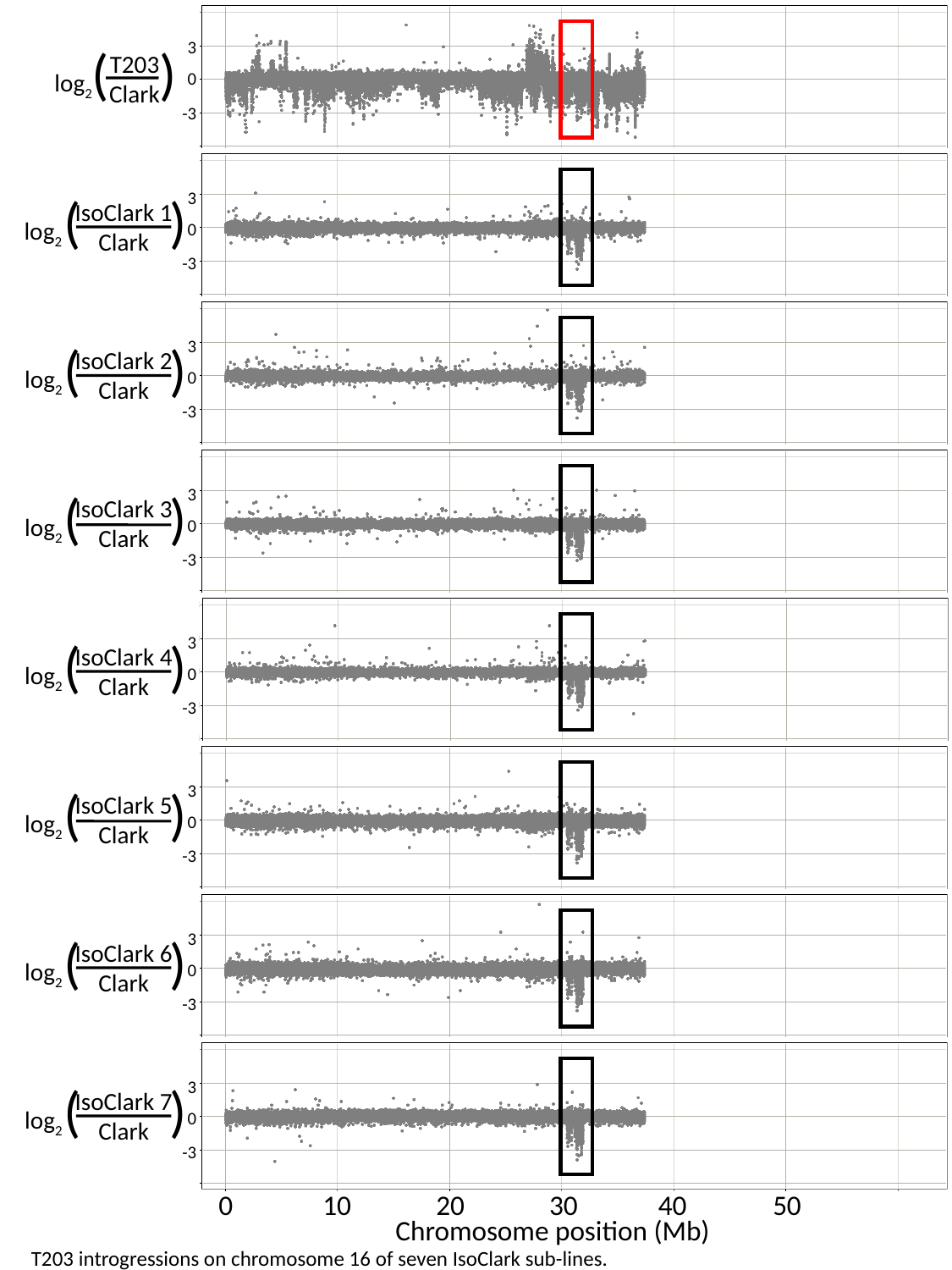

3
( )
T203
Clark
log2
0
-3
( )
3
IsoClark 1
Clark
log2
0
-3
( )
3
IsoClark 2
Clark
log2
0
-3
( )
3
IsoClark 3
Clark
log2
0
-3
( )
3
IsoClark 4
Clark
log2
0
-3
( )
3
IsoClark 5
Clark
log2
0
-3
( )
3
IsoClark 6
Clark
log2
0
-3
( )
3
IsoClark 7
Clark
log2
0
-3
0
10
20
30
40
50
Chromosome position (Mb)
T203 introgressions on chromosome 16 of seven IsoClark sub-lines.
