# Supplementary material for: Genomic Heterogeneity and Structural Variation in Soybean Near Isogenic Lines
Source: Front Plant Sci. 2013 Apr 24;4:104. doi: 10.3389/fpls.2013.00104 (PMC3633938; doi:10.3389/fpls.2013.00104)
Supplement: Supplementary Figure S2 — Examples of the 1–5 scale used for the visual scoring of IDC in the hydroponic treatments. [file Presentation_2.ppt]

## Slide 1
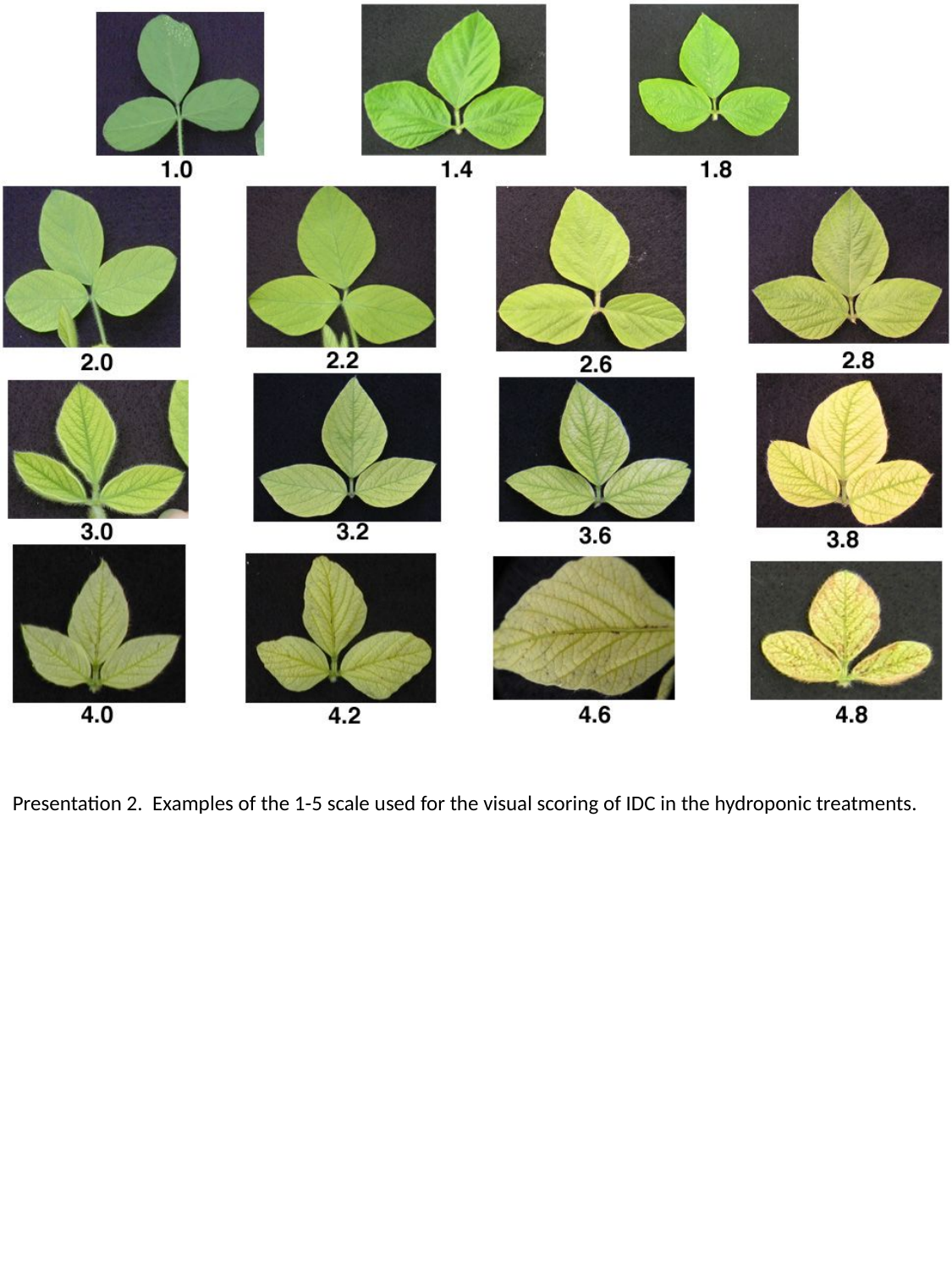

Presentation 2. Examples of the 1-5 scale used for the visual scoring of IDC in the hydroponic treatments.
